# Identification of platelet subpopulations in cryopreserved platelet components using multi-colour imaging flow cytometry

**DOI:** 10.1038/s41598-023-28352-2

**Published:** 2023-01-21

**Authors:** Lacey Johnson, Pearl Lei, Lauren Waters, Matthew P. Padula, Denese C. Marks

**Affiliations:** 1 Research and Development, Australian Red Cross Lifeblood, Alexandria, NSW Australia; 2 School of Life Sciences, University of Technology Sydney, Sydney, NSW Australia; 3 Sydney Medical School, The University of Sydney, Camperdown, NSW Australia

**Keywords:** Cell biology, Biological therapy, Fluorescence imaging

## Abstract

Cryopreservation of platelets, at  − 80 °C with 5–6% DMSO, results in externalisation of phosphatidylserine and the formation of extracellular vesicles (EVs), which may mediate their procoagulant function. The phenotypic features of procoagulant platelets overlap with other platelet subpopulations. The aim of this study was to define the phenotype of in vitro generated platelet subpopulations, and subsequently identify the subpopulations present in cryopreserved components. Fresh platelet components (n = 6 in each group) were either unstimulated as a source of resting platelets; or stimulated with thrombin and collagen to generate a mixture of aggregatory and procoagulant platelets; calcium ionophore (A23187) to generate procoagulant platelets; or ABT-737 to generate apoptotic platelets. Platelet components (n = 6) were cryopreserved with DMSO, thawed and resuspended in a unit of thawed plasma. Multi-colour panels of fluorescent antibodies and dyes were used to identify the features of subpopulations by imaging flow cytometry. A combination of annexin-V (AnnV), CD42b, and either PAC1 or CD62P was able to distinguish the four subpopulations. Cryopreserved platelets contained procoagulant platelets (AnnV^+^/PAC1^−^/CD42b^+^/CD62P^+^) and a novel population (AnnV^+^/PAC1^−^/CD42b^+^/CD62P^−^) that did not align with the phenotype of aggregatory (AnnV^−^/PAC1^+^/CD42b^+^/CD62P^+^) or apoptotic (AnnV^+^/PAC1^−^/CD42b^−^/CD62P^−^) subpopulations. These data suggests that the enhanced haemostatic potential of cryopreserved platelets may be due to the cryo-induced development of procoagulant platelets, and that additional subpopulations may exist.

## Introduction

Platelet components are transfused prophylactically to reduce the likelihood of spontaneous bleeding; or to stop active bleeding^1^. These components are conventionally stored at room-temperature (RT; 20–24 °C) with gentle agitation for up to 7 days. The short shelf-life can make inventory management challenging, sometimes leading to unavailability of fresh platelet components.

Cryopreservation offers unique advantages over storage of platelets at RT, including the extension of the platelet shelf-life to at least two years^2^. Platelet cryopreservation requires the addition of a cryoprotectant, dimethylsulfoxide (DMSO) at 5–6%, and freezing at − 80 °C^3,4^. The phenotype and functionality of thawed platelets differs significantly to conventionally stored platelets. These alterations include a reduction in the abundance of specific platelet surface receptors, externalisation of phosphatidylserine, degranulation, and shedding of extracellular vesicles (EVs)^3,5–10^. Functionally, cryopreserved platelets display enhanced thrombin generation potential with elevated peak thrombin and faster clotting times compared to RT-stored platelets^11–13^. In contrast, they have a reduced capacity to aggregate in response to agonists and adhere to collagen^7,11,14^. This divergence in function may be related to the development of platelet subpopulations within the component.

While platelets are described as being small, anucleate, discoid cells, they are not as homogenous as once thought. Subpopulations have been defined based on size, density, surface marker expression and functional responses to activation signals^15–19^. *In vitro* stimulation of platelets with both physiological and non-physiological agents, such as collagen and thrombin (C&T), A23187 and ABT-737 have also been used to investigate platelet subpopulations^17,20–25^. However, it is known that these stimulants have different potencies and conditions such as the temperature, calcium concentration, and incubation time may affect the extent of phenotypic changes observed, resulting in the formation of variable proportions of the platelet subpopulations^23,26^.

Based on these in vitro experimental models, several subpopulations (resting, aggregatory, procoagulant and apoptotic) have been assigned phenotypic attributes central to each population. Resting platelets express glycoprotein (GP)Ibα, GPVI and GPIIb/IIIa receptors, however, GPIIb/IIIa is present in the inactive conformation. Phosphatidylserine is located on the inner surface of the platelet membrane of resting platelets, and intracellularly, P-selectin is within α-granules and the mitochondrial membrane is polarised^27–29^. Aggregatory platelets promote platelet-platelet adherence and play a predominant role in fibrin clot retraction. Phenotypically, aggregatory platelets can be distinguished by the activation of GPIIb/IIIa (which binds PAC1), and degranulation resulting in P-selectin expression on the platelet surface^17,18,22,30,31^. Procoagulant platelets (alternatively called coated, necrotic, sustained calcium-induced platelets and zombie platelets) localise and accelerate thrombin generation at the site of vessel injury^18,22^. These platelets are characterised by externalisation of phosphatidylserine and depolarisation of the mitochondrial membrane^17,28,32^. They also have a lower abundance of the surface receptors associated with platelet adhesion, GPIbα and GPVI, while P-selectin is released from α-granules onto the platelet surface^17,28,30,32^. Apoptotic platelets, generated by ABT-737 stimulation, are also characterised by externalised phosphatidylserine and depolarisation of mitochondrial membranes, which align with the procoagulant phenotype^23,30,33^. ABT-737 stimulation also induces almost complete ectodomain shedding of GPIbα and GPVI, although this is time and temperature dependent^23,33^. It is important to highlight that there are still many discrepancies regarding the definition of each platelet subpopulation, as a result of the experimental design, historical definitions, and markers used to define the subpopulations and overlapping characteristics of the different subpopulations^30,32,34^. Given the absence of a single, definitive marker to distinguish procoagulant and apoptotic platelets from the other platelet subpopulations, the use of multiple characteristics in combination should facilitate a better understanding of the characteristics of the subpopulations.

Imaging flow cytometry has been successfully used to evaluate the unique characteristics of platelets^28,35–38^. This high-throughput technique combines both multi-colour flow cytometry and high-resolution imaging, via a charge-coupled device (CCD) camera. This allows quantitative analysis of the size, shape and phenotypic characteristics of platelets and EVs, from individualised microscopy images. In this study, multiple three colour fluorescence panels were designed to assess the pattern of features previously described to be determinants of platelet subpopulations. Using this protocol, we characterised the platelet subpopulations present in cryopreserved platelet components.

## Results

**Figure 1:**
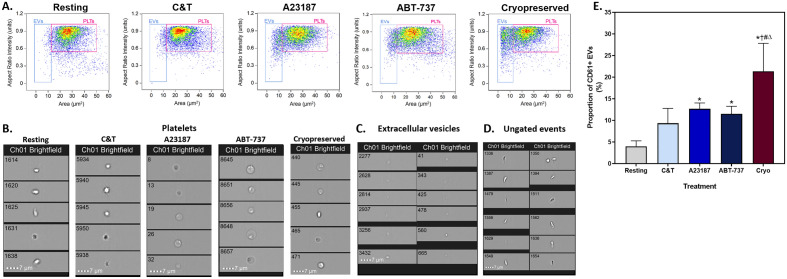
Representative scatterplots and images of platelets and extracellular vesicles. Fresh components were either unstimulated (resting), or stimulated with collagen and thrombin (C&T), A23187 or ABT-737 (n = 6 for each treatment). Cryopreserved (cryo) platelet components were thawed and reconstituted in plasma (n = 6). The populations of platelets (PLTs) and extracellular vesicles (EVs) were distinguished based on brightfield area and aspect ratio intensity. (**A**) A representative density scatterplot of each group. Representative brightfield images of (**B**) platelets, (**C**) extracellular vesicles and (**D**) ungated events. (**E**) The mean + SD (error bars) CD61^+^ EVs (% of sample) in each group. * Indicates *p* < 0.05 compared to resting; † Indicates *p* < 0.05 compared to C&T; # Indicates *p* < 0.05 compared to A23187; Δ Indicates *p* < 0.05 compared to ABT-737.

Platelet activation, apoptosis and platelet cryopreservation are known to result in the formation of EVs^8,12,28,39^. The proportion of platelet (CD61 +) EVs was increased in all treatment groups, compared to unstimulated platelets (Fig. 1), although cryopreservation generated the highest number of EVs.

**Figure 2:**
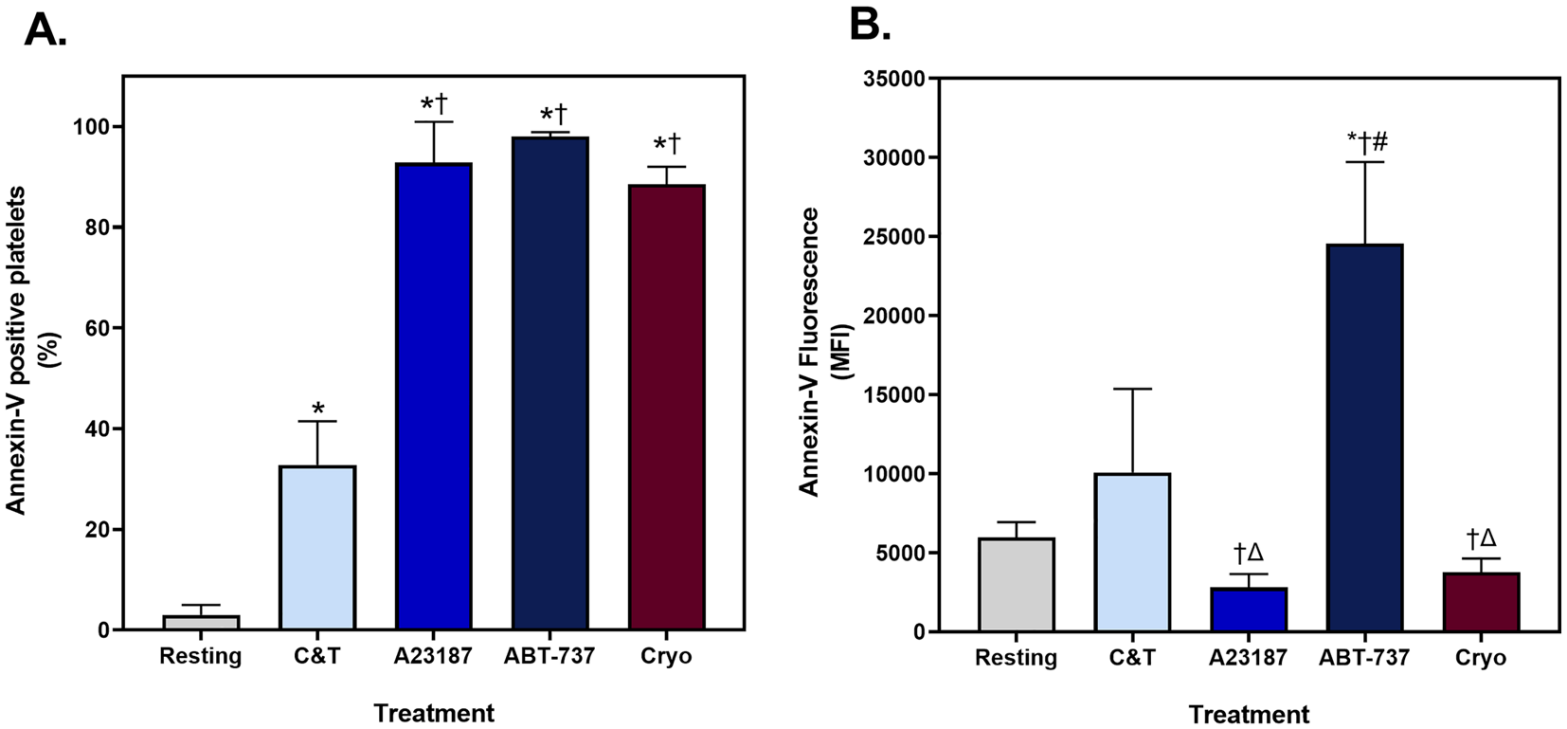
The proportion of platelets with externalised phosphatidylserine. Fresh components were either unstimulated (resting), or stimulated with collagen and thrombin (C&T), A23187 or ABT-737 (n = 6 for each treatment). Cryopreserved (cryo) platelet components were thawed and reconstituted in plasma (n = 6). Samples were analysed by imaging flow cytometry, with 7500 platelet events recorded. (**A**) The proportion of annexin-V positive (AnnV^+^) platelets and (**B**) the median fluorescence intensity (MFI) of AnnV^+^ platelets was determined from the APC fluorescence channel (Ch 05). The data represent the mean + SD (error bars). * Indicates *p* < 0.05 compared to resting; † Indicates *p* < 0.05 compared to the C&T; # Indicates *p* < 0.05 compared to A23187; Δ indicates *p* < 0.05 compared to ABT-737.

Phosphatidylserine externalisation, detected by annexin-V (AnnV) binding, allows discrimination between resting and aggregatory populations from procoagulant and apoptotic platelets^18,23,30,32^. An increase in the proportion of annexin-V positive (AnnV^+^) platelets was observed in all treatment groups, where approximately 90% of the A23187, ABT-737 and cryopreserved platelets were AnnV^+^ (Fig. 2A). In contrast, only 35% of the C&T stimulated platelets externalised phosphatidylserine, indicating multiple platelet populations were present in this group, as expected. Of interest, there was variation in the intensity of AnnV fluorescence between the treatment groups (Fig. 2B). The ABT-737 platelets displayed the highest AnnV fluorescence compared to the other treatment groups.

**Figure 3:**
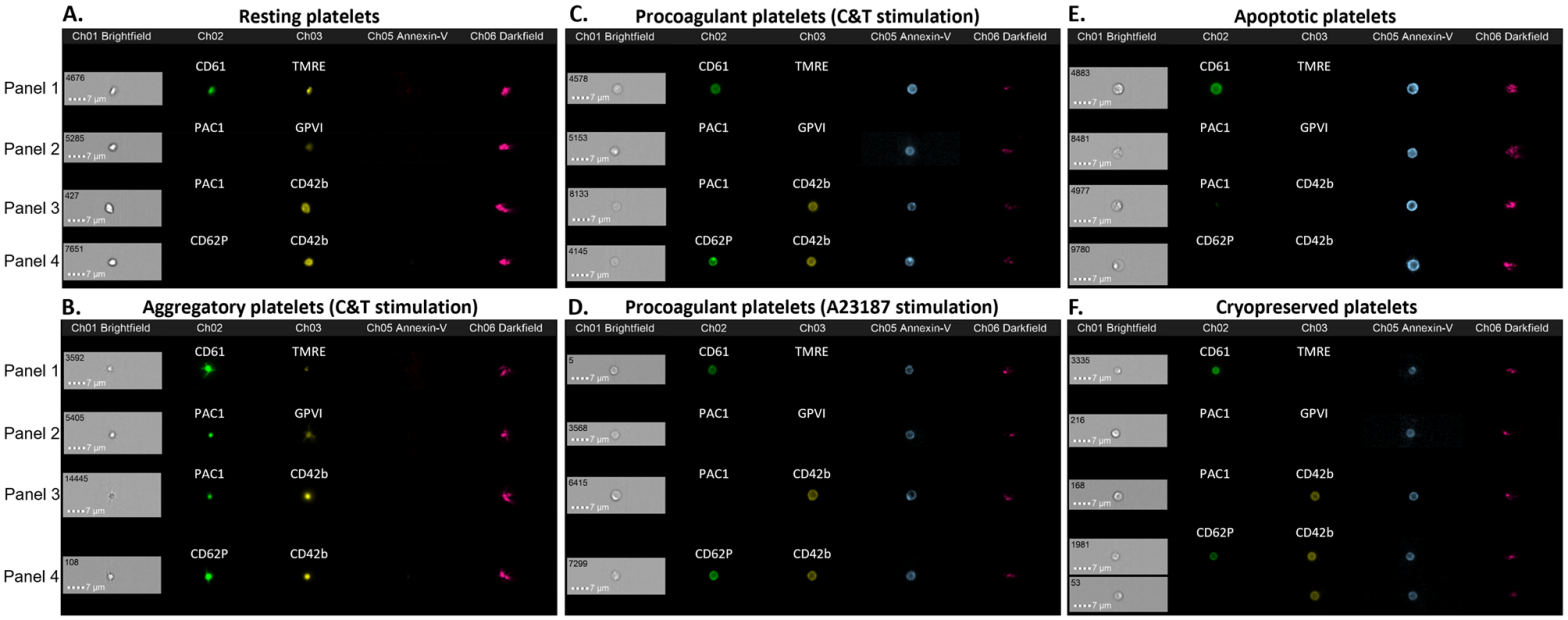
Representative images of the phenotype of platelet subpopulations within each treatment group. Fresh components were either unstimulated (resting), or stimulated with collagen and thrombin (C&T), A23187 or ABT-737 (n = 6 for each treatment). Cryopreserved platelet components were thawed and reconstituted in plasma (n = 6). The platelets were stained with four panels containing three different fluorescent antibodies/dyes and analysed by imaging flow cytometry, with 7500 platelet events recorded. Representative images of the phenotype of the most prominent platelet subpopulation(s) in the treatment group are presented. The pattern of staining observed in (**A**) resting platelets was annexin-V (AnnV^−^)/CD61^+^/TMRE^+^/PAC1^−^/GPVI^+^/CD62P^−^/CD42b^+^, (**B**) aggregatory platelets (from C&T stimulation) were AnnV^−^/CD61^+^/TMRE^+^/PAC1^+^/GPVI^+^/CD62P^+^/CD42b^+^, (**C**) procoagulant platelets (from C&T stimulation) were AnnV^+^/CD61^+^/TMRE^−^/PAC1^−^/GPVI^−^/CD62P^+^/CD42b^+^, (**D**) procoagulant platelets (from A23187 stimulation) were AnnV^+^/CD61^+^/TMRE^−^/PAC1^−^/GPVI^−^/CD62P^+^/CD42b^+^, (**E**) apoptotic platelets were AnnV^+^/CD61^+^/TMRE^−^/PAC1^−^/GPVI^−^/CD62P^−^/CD42b^−^, and (**F**) two populations of cryopreserved platelets were observed: AnnV^+^/CD61^+^/TMRE^−^/PAC1^−^/GPVI^−^/CD62P^+^/CD42b^+^ and AnnV^+^/CD61^+^/TMRE^−^/PAC1^−^/GPVI^−^/CD62P^−^/CD42b^+^.

Annexin-V was used in combination with PAC1, CD42b, GPVI, CD62P and tetramethylrhodamine ethyl ester (TMRE; to determine mitochondrial membrane polarisation status) to define the characteristics of the platelets present in each in vitro treatment group (Fig. 3). The combination of fluorescent markers in panel 1 and 2 was unable to differentiate all four subpopulations due to the overlapping features of the resting and aggregatory, and procoagulant and apoptotic subpopulations. However, panel 3 (PAC1, CD42b and AnnV) and panel 4 (CD62P, CD42b, AnnV) were able to distinguish the four platelet subpopulations of interest (Tables 1 and 2). However, the proportions of platelets in each subpopulation varied in the two panels. Representative dot-plots demonstrating the fluorescence profile of these populations are presented in Supplementary Fig. 1 online.

**Table 1:** The proportions of platelets present with the indicated triple marker phenotypes, based on annexin-V (AnnV), CD42b and PAC1.

	Fluorescent marker combination (%)							
	AnnV^−^				AnnV^+^			
Treatment group	CD42b^+^ PAC1^−^	CD42b^+^ PAC1^+^	CD42b^−^ PAC1^−^	CD42b^−^ PAC1^+^	CD42b^+^ PAC1^−^	CD42b^+^ PAC1^+^	CD42b^−^ PAC1^−^	CD42b^−^ PAC1^+^
Unstimulated	**92 ± 5**	1 ± 0	0 ± 0	1 ± 1	1 ± 0	2 ± 1	0 ± 0	0 ± 0
C&T	16 ± 7	**48 ± 17**	2 ± 1	1 ± 1	**22 ± 9**	9 ± 2	2 ± 1	0 ± 0
A23187	5 ± 7	0 ± 0	2 ± 1	1 ± 0	**88 ± 10**	1 ± 0	3 ± 4	0 ± 0
ABT-737	0 ± 0	0 ± 0	1 ± 0	0 ± 0	0 ± 0	0 ± 0	**97 ± 1**	1 ± 0
Cryopreserved	8 ± 4	2 ± 1	1 ± 0	1 ± 0	**83 ± 3**	1 ± 0	4 ± 4	0 ± 0
Subpopulation	Resting	Aggregatory	N/A	N/A	Procoagulant	N/A	Apoptotic	N/A

Data shown as mean ± SD; n = 6 in each group.

Bolded cells indicate the dominant phenotype(s) within the treatment group.

N/A indicates a minority phenotype not associated with a subpopulation.

**Table 2:** The proportions of platelets present with the indicated triple marker phenotypes, based on annexin-V (AnnV), CD42b and CD62P.

	Fluorescent marker combination (%)							
	AnnV^−^				AnnV^+^			
Treatment group	CD42b^+^ CD62P^−^	CD42b^+^ CD62P^+^	CD42b^−^ CD62P^−^	CD42b^−^ CD62P^+^	CD42b^+^ CD62P^−^	CD42b^+^ CD62P^+^	CD42b^−^ CD62P^−^	CD42b^−^ CD62P^+^
Unstimulated	**73 ± 9**	20 ± 6	1 ± 0	0 ± 0	0 ± 0	2 ± 1	0 ± 0	0 ± 0
C&T	2 ± 0	**62 ± 10**	2 ± 1	1 ± 1	3 ± 1	**29 ± 8**	1 ± 0	0 ± 0
A23187	2 ± 1	6 ± 6	1 ± 1	0 ± 0	17 ± 11	**72 ± 9**	1 ± 1	1 ± 1
ABT-737	0 ± 0	0 ± 0	1 ± 0	1 ± 0	0 ± 0	0 ± 0	**90 ± 3**	7 ± 3
Cryopreserved	6 ± 4	11 ± 5	2 ± 1	0 ± 0	**56 ± 8**	**23 ± 7**	2 ± 2	0 ± 0
Subpopulation	Resting	Aggregatory	N/A	N/A	Novel	Procoagulant	Apoptotic	N/A

Data shown as mean ± SD; n = 6 in each group.

Bolded cells indicate the dominant phenotype(s) within the treatment group.

N/A indicates a minority phenotype not associated with a subpopulation.

Novel indicates a phenotype not aligned with the known subpopulations.

As expected, the majority of platelets within fresh, unstimulated samples were annexin-V negative (AnnV^−^). The AnnV^−^ platelets had surface expression of CD61, GPIbα (CD42b) and GPVI and high TMRE fluorescence, but were negative for PAC1 and CD62P. These characteristics were used to define the resting platelet phenotype, and between 73 and 92% of platelets in the unstimulated group were resting, depending on the fluorescent marker panel (Tables 1 and 2). Representative images of the fluorescence pattern of platelets within fresh, unstimulated components are shown in Fig. 3A.

Dual stimulation of platelets with C&T is known to generate two distinct proportions of aggregatory and procoagulant platelets^22,31^. Using the panel of fluorescent markers, two populations of platelets were evident. One population did not bind AnnV, but bound PAC1, and were therefore defined as aggregatory platelets. The aggregatory platelets also stained positive for CD42b, CD62P, GPVI and maintained high TMRE fluorescence (Fig. 3B). Between 48 and 62% platelets in the C&T group had an aggregatory phenotype (Tables 1 and 2). The second population was defined as procoagulant platelets based on AnnV binding, combined with CD62P and CD42b fluorescence (Fig. 3C). The AnnV^+^ platelets were negative for PAC1 and GPVI and had little TMRE fluorescence. Between 22 and 29% of platelets in the C&T group were procoagulant (Tables 1 and 2).

Stimulation with A23187 resulted in the majority of the platelets (72–88%) acquiring a similar phenotype to that observed with the procoagulant platelets in the C&T group (Tables 1 and 2). Specifically, they were positive for AnnV, CD62P and CD42b and demonstrated loss of TMRE and GPVI fluorescence (Fig. 3D).

As expected, a high proportion of the platelets stimulated with ABT-737 were AnnV^+^. These platelets did not bind PAC1, CD42b, GPVI or CD62P and had no TMRE fluorescence (Fig. 3E). As such, AnnV^+^ platelets which were negative for the other markers were defined as being apoptotic. ABT-737 treatment resulted in between 90 and 97% of platelets acquiring an apoptotic phenotype.

Cryopreserved platelets were assessed using the same fluorescent panels. The majority of cryopreserved platelets were positive for AnnV, CD42b, CD61, and negative for PAC1, GPVI and displayed low TMRE fluorescence. The pattern of AnnV/CD42b/PAC1 staining demonstrated 83 ± 3% of the cryopreserved platelets aligned with the procoagulant phenotype (AnnV^+^/CD42b^+^/PAC1^−^; Fig. 3F; Table 1). However, assessment of CD62P staining demonstrated that a large proportion of the AnnV^+^/CD42b^+^ cryopreserved platelets did not bind CD62P, meaning that only 23 ± 7% of the platelets would be defined as procoagulant (Table 2). The phenotype of this AnnV^+^/CD42b^+^/CD62P^−^ population (novel) did not align with any of the four subpopulations currently identified.

**Figure 4:**
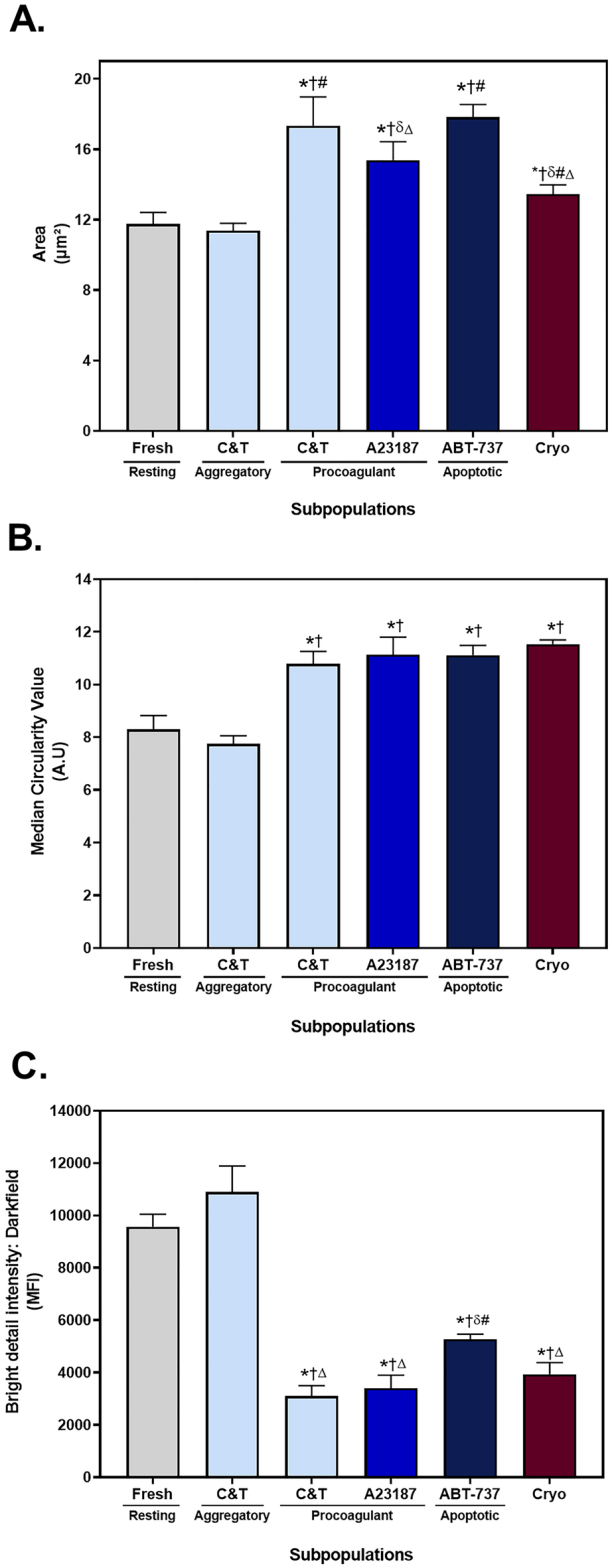
Morphological parameters of platelet subpopulations. Fresh components were either unstimulated (resting), or stimulated with collagen and thrombin (C&T), A23187 or ABT-737 (n = 6 for each treatment). Cryopreserved (cryo) platelet components were thawed and reconstituted in plasma (n = 6). Samples were analysed by imaging flow cytometry, with 7500 platelet events recorded. (**A**) The median area was calculated using a custom mask (combining the brightfield and fluorescent marker channels). (**B**) The circularity of platelets was determined using a custom mask (combining the brightfield and fluorescent marker channels). (**C**) The internal complexity of the platelets, as indicated by the median intensity of darkfield bright detail was determined using a custom mask (combining brightfield, fluorescent and darkfield channels). The data represent the mean + SD (error bar). * Indicates *p* < 0.05 compared to resting; † Indicates *p* < 0.05 compared to the aggregatory phenotype derived from C&T stimulation; δ Indicates *p* < 0.05 compared to the procoagulant phenotype derived from C&T stimulation; # Indicates *p* < 0.05 compared to A23187; Δ Indicates *p* < 0.05 compared to ABT-737.

A strength of imaging flow cytometry is that it can be used to examine and quantitate morphological features of platelets. The treatment groups were separated into the dominant subpopulations and morphological features of size, circularity and internal complexity of the platelets were assessed (Fig. 4). The mean area of fresh and aggregatory (AnnV^−^ C&T) platelets were similar (Fig. 4A). In contrast, the AnnV^+^ C&T-, A23187- and ABT-737-stimulated platelets had a larger area than the fresh platelets. Cryopreserved platelets were also larger than fresh platelets but were significantly smaller than the other AnnV^+^ platelet subpopulations. Procoagulant platelets have been associated with a circular, balloon-shaped morphology^18,28,40,41^. The fresh and aggregatory (AnnV^−^ C&T) platelets had a lower circularity value than the AnnV^+^ platelets found in the C&T-, A23187-, ABT-737-treated platelets (Fig. 4B). The cryopreserved platelets had a similar circularity value to the procoagulant platelets. The darkfield profile, equivalent to SSC, measures the internal complexity of platelets^28^, such that a low darkfield value may indicate degranulation. The darkfield intensity values of the procoagulant (AnnV^+^ C&T and A23187) and apoptotic platelets was significantly lower than the fresh and aggregatory (AnnV^−^ C&T) platelets (Fig. 4C). The darkfield intensity of cryopreserved platelets was similar to that of the procoagulant platelets, being more degranulated than apoptotic platelets. Combined, these data suggest that the morphology of cryopreserved platelets is aligned with that of procoagulant platelets.

**Figure 5:**
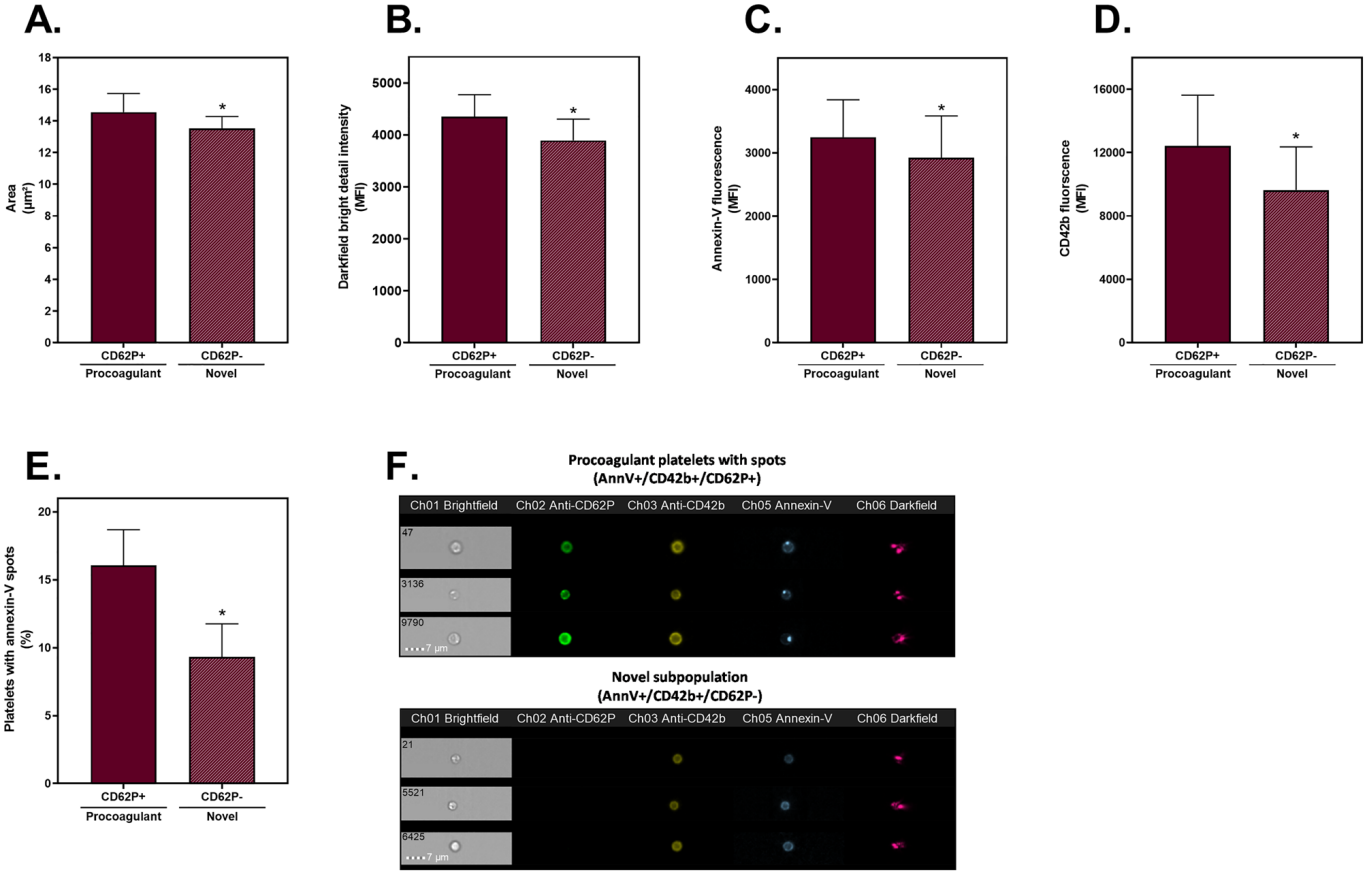
Interrogation of the CD62P^+^ and CD62P^−^ subpopulations identified in cryopreserved platelets. Cryopreserved platelet components were thawed and reconstituted in plasma (n = 6). Samples were analysed by imaging flow cytometry, with 7500 platelet events recorded. The annexin-V positive (AnnV^+^)/CD42b^+^ subpopulations which were either CD62P^+^ (procoagulant) or CD62P^−^ (novel) were compared. (**A**) The median area was calculated using a custom mask (combining the brightfield and fluorescent marker channels). (**B**) The median intensity of darkfield bright detail was determined using a custom mask (combining brightfield, fluorescent and darkfield channels). The median fluorescence intensity (MFI) of (**C**) AnnV and (**D**) CD42b of the positive platelets. (**E**) The proportion of each subpopulation containing AnnV^+^ spots was calculated using the spot count feature, with (**F**) representative images of CD62P^+^ and CD62P^−^ cryopreserved platelets displaying the presence and absence of AnnV^+^ spots. The data represent the mean + SD (error bar). * Indicates *p* < 0.05 compared to CD62P^+^ cryopreserved platelets.

Focusing on the cryopreserved group, the morphological parameters were combined with the fluorescent markers in an effort to understand the differences between the AnnV^+^/CD42b^+^/CD62P^+^ and CD62P^−^ populations. Based on area and darkfield (SSC), the AnnV^+^/CD42b^+^/CD62P^−^ platelets were smaller and had less internal complexity than the procoagulant CD62P^+^ platelets (Fig. 5A and B). Further, the fluorescence intensity of the AnnV and CD42b staining was lower in the CD62P^−^ platelets than the CD62P^+^ platelets in the cryopreserved group (Fig. 5C and D). The staining pattern of the platelets was also assessed and the presence of bright spots of AnnV fluorescence was identified. These spots have previously been referred to as platelet-associated EVs^28^. The proportion of platelets with AnnV^+^ spots was almost twofold lower in the CD62P^−^ group, compared to the CD62P^+^ cryopreserved platelets (Fig. 5E and F). This may arise due to release of EVs from the CD62P^−^ platelets, while the EVs in the CD62P^+^ platelets are still attached.

## Discussion

Cryopreserved platelet components were first introduced in military settings due to the reduced access to fresh platelet components^2,42,43^. The extended shelf-life of cryopreserved platelet components is advantageous for inventory management and allows stock-piling^43^. Furthermore, cryopreserved components have been shown to be clinically safe and potentially have enhanced haemostatic function in actively bleeding patients^43–48^. Although the exact mechanism mediating the therapeutic benefit is incompletely understood, the changes induced by cryopreservation seem to enhance their procoagulant potential^10–12,14^.

Currently, the definition of procoagulant platelets is ambiguous because the term encompasses many physiologically- and non-physiologically in vitro-induced platelet populations, with phosphatidylserine externalisation as a key feature^20–24^. However, this marker is also the primary delineator of apoptotic platelets^23,34,49^. Further, there is no individual feature that can distinguish procoagulant platelets from other platelet subpopulations^28,34,50^. To date, few studies have directly compared the profile of the four most commonly described platelet subpopulations (resting, aggregatory, procoagulant and apoptotic)^26^, and no study has focused on defining the proportions of these subpopulations in ex vivo stored platelet components. The results presented here define the co-existing features present on individual platelets, using multi-colour imaging flow cytometry, in an effort to provide a more robust definition of these subpopulations. The proportions of platelets present in each treatment group were aligned with what would be expected based on the published literature^20,25,26,28,51^, giving confidence that the correct phenotype had been identified. These definitions then facilitated identification of the platelet subpopulations present in cryopreserved components.

Aggregatory platelets were only observed following strong stimulation with C&T, and could be distinguished from the other platelet subpopulations, as they were the only subpopulation that expressed the activated GPIIb/IIIa receptor (PAC1^+^). In this, and other studies, cryopreserved platelets bind little PAC1^7,10,14,52^, suggesting GPIIb/IIIa is inactive and aggregatory platelets are not abundant in cryopreserved components. This aligns with the functional findings that cryopreserved platelets have a diminished capacity to aggregate in vitro in response to agonist stimulation^7,11,53^. However, it has also been hypothesised that loss of PAC1 binding may be due to the GPIIb/IIIa receptor being engaged by fibrinogen or an alternative ligand with greater affinity than PAC1 ^20^. This is yet to be determined for cryopreserved platelets.

The AnnV^+^ platelets generated by strong agonist stimulation (C&T) and A23187 had the accompanying characteristic procoagulant features of balloon morphology, degranulation and externalised P-selectin (CD62P^+^), and mitochondrial membrane depolarisation ^17,28,30,32^. GPVI was absent, and although the MFI of GPIbα was lower compared to resting platelets, all platelets remained CD42b^+^ (Supplementary Fig. S1 online). In the literature, A23187 has been used to generate activated, procoagulant and apoptotic platelets ^24,25,28^, although the assignment of different nomenclature has likely arisen from differences in the research focus and design of the studies. In our study, platelets generated by A23187 stimulation aligned both phenotypically and morphologically with the procoagulant platelets generated by dual C&T treatment, rather than apoptotic platelets. This suggests, that at least under these conditions, A23187 treatment provides a good model to achieve a relatively pure population of procoagulant platelets.

The majority of the ABT-737-treated platelets acquired features that have previously been associated with apoptosis ^23,26,33^. Specifically, they were AnnV^+^, and negative for all the other markers tested in this study. The absence of CD42b and CD62P on apoptotic platelets (AnnV^+^/CD42b^−^/CD62P^−^) facilitated separation from the procoagulant platelet (AnnV^+^/CD42b^+^/CD62P^+^) population. While CD62P is often used to distinguish procoagulant from apoptotic platelets ^26,50^, the use of this marker without CD42b would have led to a large proportion of the cryopreserved platelets being defined as apoptotic (AnnV^+^/CD62P^−^). Further, while it is known that treatment of ABT-737 is time, temperature and calcium dependant ^26^, it is important to note an extended ABT-737 treatment time (4 h) was required for the majority of the platelets to become CD62P^−^. In contrast, all the AnnV^+^ platelets were negative for other markers within 2 h (Supplementary Table S1 online). As such, the use of CD62P as a marker of apoptosis should only be considered in combination with additional markers of procoagulant platelets. Importantly, while a proportion of the cryopreserved platelets do not express CD62P, they still express GPIbα (CD42b^+^), albeit at a reduced abundance, and they were morphologically distinct from the apoptotic phenotype generated throughout ABT-737 stimulation.

Cryopreserved platelets undergo marked morphological and phenotypic changes, and previous data has indicated that post-thaw platelets are heterogenous^9,10,54^. A proportion of these platelets align with the traditional procoagulant phenotype, including externalised phosphatidylserine, CD42b and CD62P surface expression, inactive GPIIb/IIIa and depolarised mitochondrial membrane. Although smaller than the procoagulant platelets generated by in vitro stimulation, cryopreserved platelets also had a high circularity index and demonstrate the balloon shape characteristic of procoagulant platelets, which is thought to contribute to thrombin generation, and may be a novel mechanism for EV generation^40^.

Of interest, a subpopulation of platelets that did not express surface CD62P was also identified in cryopreserved components. These CD62P^−^ platelets were smaller, more degranulated and expressed a lower abundance of GPIbα and phosphatidylserine on their surface than the procoagulant (CD62P^+^) platelets. Taken together, the results suggest that CD62P is likely being removed from the surface, via cleavage, rather than never being externalised. We have previously shown that soluble CD62P increases in the supernatant of cryopreserved platelets, in a time dependent manner, suggesting cleavage may occur^6,54^. However, combined with the loss of area and other surface markers, the CD62P^−^ platelets may result from EV formation. This hypothesis is also supported by the reduction in platelet-associated EVs (AnnV^+^ spots) present on the CD62P^−^ platelets, and this aligns with the known increase in EVs present in cryopreserved components^3,8,12^. Further, the disintegration of fragile balloon structures may generate large numbers of EVs^55,56^. Of note, the AnnV^+^/CD42b^+^/CD62P^−^ population observed in the cryopreserved platelets was also evident in the A23187-treated platelets, albeit at a lower proportion (17 ± 11%; Table 2), so is not unique to the cryopreservation process. This suggests that there may be a transitional subpopulation generated beyond the procoagulant state, perhaps related to the formation of EVs.

The current literature proposes that the development of platelet subpopulations may be based on a spectrum of activation, depending on the strength of stimulation and the cytoplasmic calcium concentration^57,58^. It has also been suggested that procoagulant and apoptotic platelets may be interrelated^24,25,34^. Jackson et al*.*, suggest that procoagulant platelets are actually undergoing cell death^34^; referring to them as necrotic cells^34,50^. However, it is clear that there are knowledge gaps in the current understanding of platelet phenotypes, and there is a growing body of evidence suggesting additional distinct platelet subpopulations may exist^59–62^.

While many previous studies have investigated the functional capacity of cryopreserved platelets^9,12,14,45,63^, it will be important to specifically determine the functional capacity of the AnnV^+^/CD42b^+^/CD62P^−^ population identified in this study. Given that cryopreserved platelet components contain a high proportion of this population, it may contribute to their procoagulant activity. Of note, the CD62P^−^ population were smaller than the traditional procoagulant platelets, and we have previously shown that the smaller platelets generated by cryopreservation are associated with enhanced clot formation using viscoelastic testing (R-time)^54^. Importantly, Michelson et al*.*^64^ have demonstrated that degranulated CD62P^−^ platelets continue to circulate and function haemostatically in vivo. However, it is also necessary to consider this subpopulation in the context of non-haemostatic functions of platelets, as P-selectin (CD62P) is the key mediator of leukocyte interactions^65^.

In summary, multi-colour imaging flow cytometry was used to identify distinct platelet subpopulations within the thawed cryopreserved platelet component. As anticipated, a proportion of these platelets were morphologically and phenotypically aligned with traditional procoagulant platelets. However, the more dominant population likely represent an intermediate population resulting from degranulation and EV release from procoagulant platelets. The significance of this population remains to be determined, and further investigation is warranted to assess the functional potential and circulatory capability of these platelets once transfused.

## Methods

Ethics approval was obtained from the Australian Red Cross Lifeblood Human Research Ethics Committee. Informed consent was obtained from donors for all blood donations used in this study. Donations were collected from eligible, voluntary donors, in accordance with Lifeblood standard operating procedures.

Buffy coat-derived platelet components were prepared in 30% plasma/70% platelet additive solution (PAS-E; SPP +, Macopharma, Mouvaux, France), as previously described^4^. A sample (5 mL) was removed from the component via sterile transfer for testing/stimulation, and then the remainder of the component was cryopreserved, as described below. The platelet count was determined using an automated haematology analyser (CELL DYN Emerald, Abbott Diagnostics, IL, USA) to calculate the necessary dilutions.

### In vitro stimulation to generate platelet subpopulations

**Figure 6:**
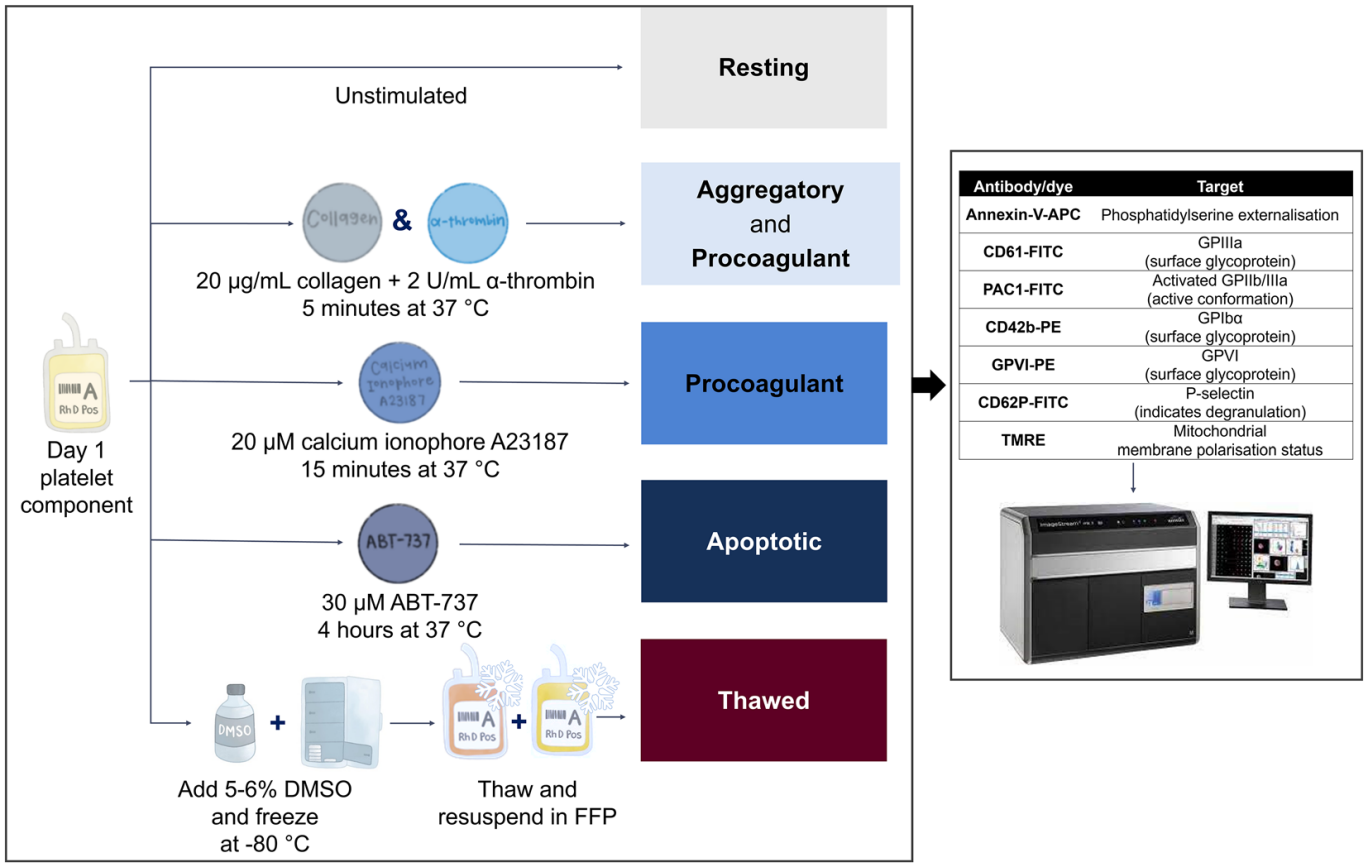
The experimental design for generation of platelet subpopulations. Fresh platelet components were either unstimulated (resting) or stimulated with collagen and α-thrombin (C&T), calcium ionophore A23187 or ABT-737 (n = 6 for each treatment). Stimulation with these agents is known to induce aggregatory and procoagulant (C&T), procoagulant (A23187) or apoptotic (ABT-737) platelets, respectively. Fresh platelet components (n = 6) were frozen at  − 80 °C with 5–6% DMSO. Prior to testing, cryopreserved platelet components were thawed at 37 °C and reconstituted in fresh frozen plasma (FFP). Samples from each group were stained with multiple fluorescent markers, as indicated, and data were acquired using an imaging flow cytometer (ImageStream^X^ Mark II) and then analysed using IDEAS software.

For all stimulation and staining, the platelets were diluted in annexin-V (AnnV) binding buffer (Biolegend, San Diego, CA, USA), to provide a source of extracellular calcium. Fresh platelets (day 1 of ex vivo storage) were either left unstimulated or stimulated (C&T, calcium ionophore A23187 and ABT-737) to generate the desired subpopulations, using methods previously described in the literature. Following this, the remainder of the platelet component was frozen and thawed. The experimental design is summarised in Fig. 6. The characteristics of platelets within the treatment groups was used to define each platelet subpopulation.

Dual stimulation with the physiological agonists, collagen and thrombin (C&T) has been used to generate different proportions of procoagulant and aggregatory platelets^20,29^. Fresh platelets were pre-incubated with 2.5 mM Gly-Pro-Arg-Pro (GPRP; Sigma-Aldrich, St Louis, MO, USA) for 15 min at 37 °C. GPRP prevents the polymerisation of fibrinogen, and thus, reduces thrombin-induced platelet aggregation. The platelet sample was then stimulated with 20 µg/mL collagen (Helena Laboratories, Beaumont, TX, USA) and 2 U/mL α-thrombin (Enzyme Research Labs, South Bend, IN, USA) for 5 min at 37 °C^66^. The reaction was stopped by the addition of filtered AnnV binding buffer.

Calcium ionophore (A23187) facilitates increased calcium entry and has been used to generate both procoagulant and apoptotic populations, depending on the research focus of the study^24,25,28^. Fresh platelets were stimulated with 2 µM A23187 (Sigma-Aldrich) for 15 min at 37 °C^26,67^.

ABT-737 is a non-physiological agent that has been used in both human and mouse models to understand platelet apoptosis, as it inhibits anti-apoptotic BCL-2 protein family regulators^24,49^. Apoptotic platelets were generated by stimulating fresh platelets with 30 µM ABT-737 (SelleckChemicals, Houston, TX, USA) for 4 h at 37 °C.

### Cryopreservation and thawing

For cryopreservation, approximately 100 mL of 27% wt/vol DMSO/0.9% saline (Sypharma Pty. Ltd, Dandenong, VIC, Australia) was added to each platelet component to achieve a final concentration of 5–6% (v/v)^4^. The platelets were centrifuged, and the DMSO-containing supernatant was removed. The hyperconcentrated platelets were then frozen at −80 °C, where they were stored for a minimum of one week prior to thawing.

Platelets were thawed in a water bath at 37 °C. Thawed platelets were then reconstituted in freshly thawed plasma (250–310 mL)^4^. The thawed plasma was joined to the thawed platelet component, using a sterile welder, and the platelets were resuspended in the plasma with gentle circular agitation. The platelets were sampled immediately, counted and diluted in AnnV binding buffer as for fresh platelets.

### Imaging flow cytometry

Samples were taken from each of the five groups after stimulation/thawing for analysis by imaging flow cytometry. Platelets were diluted to 10 × 10^6^/mL in 0.1 µm PVDF filtered calcium-containing AnnV binding buffer and 50 µL was stained with a panel of three fluorescent markers in multiple combinations. All tubes contained annexin-V-allophycocyanin (APC; 1 µL neat) to allow the comparison of fluorescent markers across the four staining panels. Platelet samples were labelled with 1 µL of the following fluorescent antibodies and/or dyes: Panel 1: CD61-fluorescein isothiocyanate (FITC), tetramethylrhodamine ethyl ester (TMRE), AnnV-APC; Panel 2: PAC1-FITC, GPVI-phycoerythrin (PE), AnnV-APC; Panel 3: PAC1-FITC, CD42b-PE, AnnV-APC; Panel 4: CD62P-FITC, CD42b-PE, AnnV-APC. The fluorescent markers were diluted, as indicated in Supplementary Table S2 online, to maximise fluorescence, while avoiding fluorophore saturation.

Data were collected using an Amnis ImageStream^X^ Mark II (IS^X^_,_ EMD Millipore, Seattle, WA, USA) multi-spectral imaging flow cytometer using the INSPIRE acquisition software (Luminex, Seattle, WA, USA). Images were acquired using a single-camera system with brightfield illumination and three excitation lasers (488, 642 and 785 nm), with 60 × magnification on low flow rate/high sensitivity. The laser power was set on the raw maximum pixel values of the samples to ensure sufficient fluorescence signals were recorded without saturation. The laser powers were maintained at equivalent levels throughout the experiments using the calibration process. Brightfield was collected in channel 1, darkfield (side scatter) was collected in channel 6, AnnV-APC was collected in channel 5, and the fluorescence of other targets was detected in the relevant channels: FITC-conjugated markers (CD61, PAC1, CD62P) were collected in channel 2, while PE-conjugated markers (CD42b and GPVI) and TMRE were collected in channel 3. Speed beads were used during the start-up and sample acquisition processes to set the detection thresholds for optimised and consistent instrument performance. Single-stained controls were used to calculate compensation matrices, and relevant fluorescence minus one (FMO) and biological comparison controls were used to discriminate the negative and positive gates for each fluorescent marker. For collection, platelets were defined based on area vs aspect ratio intensity, and a target of 7500 platelet events were collected for each sample.

Captured images were analysed and optimised (fluorescent gradient RMS, 42–79) using Image Data Exploration and Analysis Software (IDEAS; Luminex, Seattle, WA, USA). The gating strategy used for analysis was based on previously published literature^28,38^. Platelets and EVs were identified based on CD61 positivity. Density plots of area (brightfield; in pixels) versus aspect ratio intensity (brightfield) were then used to identify and gate platelet and EV populations. Platelets were defined as events with an aspect ratio of 0.55–1.026 and an area of 12.57–50 µm^2^. EVs were defined as events with an aspect ratio intensity of less than 1.026 and an area of less than 12.57 µm^2^. Irregularly-shaped objects with an aspect ratio intensity < 0.55, and an area > 12.57 μm^2^, including platelet doublets, small aggregates and other debris were excluded from the analysis (ungated; Fig. 1). Platelets were examined according to marker fluorescence intensity, as well as features in the size, shape and texture categories. Area and circularity measurements were based on a custom mask (combining masks of brightfield (adaptive erosion coefficient, 80) and channel 02, 03 and 05), while bright detail intensity was based on darkfield. Platelets containing spots of high-fluorescence were detected using the spot count feature. A “truth population” of least 20 platelets containing AnnV^+^ spots were manually identified, to allow the software to subsequently enumerate this population in an automated manner.

Co-existing features were used to classify the platelet subpopulations. To do this, the AnnV^−^ or AnnV^+^ populations were identified, and then the proportion of platelets with each triple fluorescence profile was analysed. The expression of surface receptors and activation markers in the fresh and stimulated groups were then aligned with the anticipated phenotypes, as reported in the existing literature^23,25,26,28,31,39^, to classify into the most likely platelet subpopulation(s).

### Statistical analysis

The data were analysed using GraphPad Prism 9.4.1 (GraphPad Software Inc.; La Jolla, CA, USA) and results are expressed as mean ± standard deviation (SD). Repeated measures one-way analysis of variance (ANOVA) and *post-hoc* Bonferroni multiple comparisons test or paired two-sided t-tests were used to assess differences between the groups, as appropriate. A *p*-value of less than 0.05 was considered to be significant.

## Supplementary Information

Supplementary Information.

## Data Availability

The datasets generated during and/or analysed during the current study are available from the corresponding author on reasonable request.
