# Supplementary material for: Identification of platelet subpopulations in cryopreserved platelet components using multi-colour imaging flow cytometry
Source: Sci Rep. 2023 Jan 21;13:1221. doi: 10.1038/s41598-023-28352-2 (PMC9867743; doi:10.1038/s41598-023-28352-2)
Supplement: Supplementary file 1 — Supplementary Information. [file 41598_2023_28352_MOESM1_ESM.pdf]

Identification of platelet subpopulations in cryopreserved platelet components using multi-colour imaging flow cytometry

Lacey Johnson[1,2,*], Pearl Lei[1,2], Lauren Waters[1], Matthew P. Padula[2], Denese C. Marks[1,3]

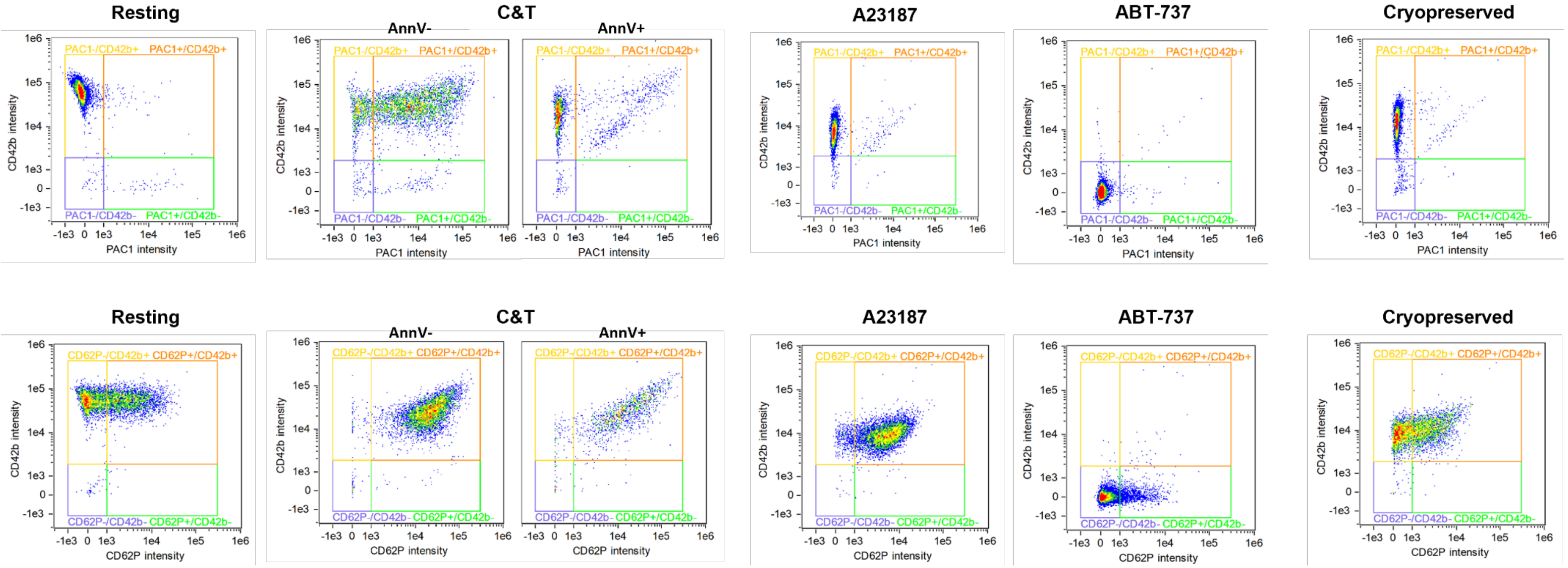

Supplementary Fig. S1. Representative scatterplots of platelet subpopulations from the five treatment groups. Fresh components were either unstimulated (resting), or stimulated with collagen and thrombin (C&T), A23187 or ABT-737. Cryopreserved platelet components were thawed and reconstituted in plasma. The platelets were distinguished based on brightfield area and aspect ratio intensity and then the subpopulations were defined based on annexin-V (AnnV), PAC1 and CD42b (upper panels) and annexin-V, CD62P and CD42b (lower panels). Representative images of the fluorescence profile of platelet population in the treatment groups are presented.

Supplementary Table S1. Time course of phenotype following ABT-737 stimulation.

	% subpopulations							
	AnnV-				AnnV+			
**ABT-737 treatment**	**CD42b$^+$ CD62P$^-$**	**CD42b$^+$ CD62P$^+$**	**CD42b$^-$ CD62P$^-$**	**CD42b$^-$ CD62P$^+$**	**CD42b$^+$ CD62P$^-$**	**CD42b$^+$ CD62P$^+$**	**CD42b$^-$ CD62P$^-$**	**CD42b$^-$ CD62P$^+$**
2 hours	0.4	0.2	5.7	1.7	1.8	2.4	70.2	17.2
4 hours	0.2	0.0	1.4	0.4	0.2	0.2	90.3	7.1
Subpopulation	**Resting**	**Aggregatory**	N/A	N/A	**Novel**	**Procoagulant**	**Apoptotic**	N/A

Shaded cells indicate the dominant phenotype(s) within the treatment group

N/A indicates a minority phenotype not associated with a subpopulation

Novel indicates a phenotype not aligned with the known subpopulations

Supplementary Table S2. Antibodies and dyes used for imaging flow cytometry

Antibody/dye	Target	Manufacturer	Catalogue number	Clone	Stock concentration	Dilution prior to adding to staining tube	Volume added to 50 µL staining tube (µL)
Annexin-V-APC	Phosphatidylserine externalisation	Biolegend, San Diego, CA, USA	640941	N/A	8 µg/mL	N/A	1
CD61-FITC	GPIIIa (surface glycoprotein)	Dako, Glostrup, Denmark	F0803	Y2/51	0.1 µg/mL	1/20	1
CD41/CD61-FITC	Activated GPIIb/IIIa (activation marker)	BD Biosciences, San Jose, CA, USA	340507	PAC-1	25 µg/mL	N/A	1
CD42b-PE	GPIbα (surface glycoprotein)	BD Biosciences, San Jose, CA, USA	555473	HIP1	25 µg/mL	N/A	1
GPVI-PE	GPVI (surface glycoprotein)	BD Biosciences, San Jose, CA, USA	565241	HY101	50 µg/mL	1/2	1
CD62P-FITC	Exposed P-selectin (activation marker)	BD Biosciences, San Jose, CA, USA	550866	AC1.2	50 µg/mL	N/A	1
TMRE	Mitochondrial membrane polarisation status	Abcam, Cambridge, United Kingdom	ab113852	N/A	1 mM	1/200	1

Abbreviations: allophycocyanin (APC); fluorescein isothiocyanate (FITC); no dilution was performed (N/A); phycoerythrin (PE); tetramethylrhodamine ethyl ester (TMRE).
